# Natural killer cell-related gene signature predicts malignancy of glioma and the survival of patients

**DOI:** 10.1186/s12885-022-09230-y

**Published:** 2022-03-02

**Authors:** Chenglong Li, Fangkun Liu, Lunquan Sun, Zhixiong Liu, Yu Zeng

**Affiliations:** 1 Department of Neurosurgery, Xiangya Hospital, Central South University, 87 Xiangya Road, Changsha, 410008 Hunan China; 2 National Clinical Research Center for Geriatric Disorders, Xiangya Hospital, Central South University, 87 Xiangya Road, Changsha, 410008 Hunan China; 3 Center for Molecular Medicine, Xiangya Hospital, Central South University, 87 Xiangya Road, Changsha, 410008 Hunan China

**Keywords:** Glioma, Natural killer cell, Signature, Prognosis, Brain tumor

## Abstract

**Background:**

Natural killer (NK) cells-based therapies are one of the most promising strategies against cancer. The aim of this study is to investigate the natural killer cell related genes and its prognostic value in glioma.

**Methods:**

The Chinese Glioma Genome Atlas (CGGA) was used to develop the natural killer cell-related signature. Risk score was built by multivariate Cox proportional hazards model. A cohort of 326 glioma samples with whole transcriptome expression data from the CGGA database was included for discovery. The Cancer Genome Atlas (TCGA) datasets was used for validation. GO and KEGG were used to reveal the biological process and function associated with the natural killer cell-related signature. We also collected the clinical pathological features of patients with gliomas to analyze the association with tumor malignancy and patients’ survival.

**Results:**

We screened for NK-related genes to build a prognostic signature, and identified the risk score based on the signature. We found that NK-related risk score was independent of various clinical factors. Nature-killer cell gene expression is correlated with clinicopathological features of gliomas. Innovatively, we demonstrated the tight relation between the risk score and immune checkpoints, and found NK-related risk score combined with PD1/PDL1 patients could predict the patient outcome.

**Conclusion:**

Natural killer cell-related gene signature can predict malignancy of glioma and the survival of patients, these results might provide new view for the research of glioma malignancy and individual immunotherapy.

**Supplementary Information:**

The online version contains supplementary material available at 10.1186/s12885-022-09230-y.

## Introduction

Glioma is the most common and malignant primary brain tumor, the prognosis of glioma patients varies greatly and mainly depends on the clinical characteristics [1]. Glioblastoma multiforme (GBM) is the most lethal and malignant brain tumor, the median overall survival of GBM is around 15 months despite surgery and combined radio- and chemo-therapy [1]. Recently, increasing evidence showed that immune infiltration was correlated with the prognosis of the glioma, precise therapies like target therapy and immunotherapy are promising ways to treat GBM. Immune-checkpoint inhibitors, Chimeric antigen receptor (CAR) T cell therapy, Natural killer cell-related therapies, Virotherapy, and Dendritic cells (DC) vaccination were the most encouraging areas in GBM therapy [2].

The molecular classification of central nervous system tumors in 2016 remarkably improved the diagnosis and prognosis prediction by IDH, MGMT methylation, TERT, TP53 et al. [3]. However, more precise signatures are needed. Recently, a few gene expression-based risk signatures in autophagy, hypoxia, ferroptosis, and glucose has explored their value in predicting prognosis of glioma patients [4–7]. Construction of the immune signature in the survival and malignancy prediction of glioma may lead to a more complete understanding of tumor microenvironment and immunotherapy.

Natural killer(NK) cells are innate cytotoxic lymphocytes encompassing distinct populations based on CD56 intensity in humans and involved in the surveillance and elimination of cancer [8]. NK cells could recognize the major histocompatibility complex (MHC) molecules, and kill target cells if they lack of MHC molecules on their surface. As one of the most cutting-edge immunotherapeutic strategies, NK cells related therapies such as adoptive NK cell transfer, chimeric antigen receptor-expressing NK cells (CAR-NKs), bispecific and trispecific killer cell engagers (BiKEs and TriKEs) have emerged as a promising therapeutic target in glioma [9], breast cancer [10], lung cancer [11], colon cancer [12], prostate cancer [13] and hematological malignancies [14]. Although great progress has been made for natural killer cell-based therapy in preclinical and clinical research, there are many things we need to do to advance the research. For instance, we have learned how NK cells employ to recognize and eliminate tumor cells and how cancer cells can also educate and evade NK cell responses [11, 15], little is known about NK cells postsurgical dysfunction and why it works well in hematological malignancies while not good enough for solid tumors [16].

A couple of studies have investigated that the NK cells were one of the least numerous immune cell populations infiltrating the tumour. They represent around 2.11% of the total and the most abundant phenotype is CD56^dim^CD16^neg^ [17]. Surprisingly, those limited NK cells were potent effectors against brain tumor. Lee SJ et al. showed that human NK cells had a strong effect against GBM and could prevent systemic metastasis of GBM [18]. Mukherjee S et al. [19] demonstrated that curcumin phytosome induced natural killer cell-dependent repolarization of GBM tumor-associated microglia/macrophages to kill GBM and their stem cells. Scientists also found that virotherapy is limited partially by an antiviral NK cell response involving specific natural cytotoxicity receptors to enhance GBM virotherapy [9].

The prognostic significance of NK cells’ activity has been demonstrated in patients with a few solid tumours [20]. NK cells signature has been found to be a determinant indicator for pathological response and extended overall survival in post therapy advanced rectal cancer patients [21]. A score system which was assessed by 10 genes related to NK cells significantly revealed the heterogeneity within the stage IV colorectal patients, warranted the importance of further stratification of those patients [22]. Ombretta Melaiu et al. demonstrate that NK cells and DC related gene signatures were not only strongly correlated with the expression of PD-1 and PD-L1 but also able to predict prognosis of neuroblastoma patients [23].

However, little is known about NK cells signature in the malignancy and prognosis in glioma [24]. In the current study, we screened for NK-related genes and built a prognostic signature. Univariate and Multivariate Cox regression analysis was applied to identify and verify the risk score based on the signature. We analyze the NK-related risk score and various clinical factors, including age, sex, IDH1 mutation, and GBM subtype, etc. GO and KEGG were used to reveal the biological process and function associated with the natural killer cell-related signature. Innovatively, we combined the risk score with immune checkpoints to sort out the glioma patients for patient prognosis prediction.

## Materials and methods

### Data collection

The mRNA sequencing data of genes encoding calmodulin dependent proteins was downloaded from The Cancer Genome Atlas (TCGA) dataset which was set as the training cohort. The mRNA sequencing data from The Chinese Glioma Genome Atlas (CGGA) dataset was set as the validation cohort. Corresponding clinical information was also downloaded.

### Gene signature building

We downloaded 134 NK cell related genes from immport (https://www.immport.org/resource) and 18 NATURAL_KILLER_CELL related GO pathways from MSigDB (The Molecular Signatures Database) [25]. After eliminating duplicates in the two databases, a total of 244 genes were ready for analysis. Univariate Cox analysis was firstly performed via R package ‘survival’, and genes with *P* values less than or equal to 0.1 were retained. To assess whether the risk score is independent of other clinical factors, multivariable Cox proportion hazard regression models were performed with the R package ‘glmnet’ [26, 27]. By combining the rank of *p* values of the univariate Cox regression analysis and Kaplan-Meier method in CGGA, three genes (FDR < 0.05) were retained to developed risk score as a linear combination of the gene expression level (expr) weighted by the regression coefficients (Coeffs) derived from the univariate Cox regression analysis. The risk score for each individual was calculated as follows:

$$\mathrm{Risk}\kern0.17em \mathrm{score}=\exp {\mathrm{r}}_{\mathrm{gene}1}\times {\upbeta}_{\mathrm{gene}1}+{\mathrm{expr}}_{\mathrm{gene}2}\times {\upbeta}_{\mathrm{gene}2}+\dots +{\mathrm{expr}}_{\mathrm{gene}\mathrm{n}}\times {\upbeta}_{\mathrm{gene}\mathrm{n}}$$

We determined cutoff points to significantly split (log-rank test *P* value < 0.05) the training group into low/high risk score groups [28]. The same β_genen_ that represents the coefficient of the corresponding gene was applied to the validation cohort.

### GO and KEGG pathway analyses of DEGs

The genes between NK cell-related high-risk and low-risk groups were screened via the R package ‘limma’. The gene with a absolute value of log2 fold change (FC) > 1 and adjusted *p* < 0.05 was identified as DEG. R languagt ‘clusterProfiler’, ‘colorspace’, and ‘enrichplot’ package was usd to perform GO and KEGG analysis. GO analysis with functions including molecular function (MF), biological pathways (BP), cellular component (CC), and KEGG pathway analyses were performed to the DEGs. Kaplan–Meier plots and the log-rank test used to estimate the survival rate between the low- and high-risk groups by using R package ‘survminer’ [29]. *P* < 0.05 and q < 0.05 were considered to have a significance.

### Statistical analysis

R language (version 3.5.0, https://www.r-project.org/) was the main tool for data analysis and figure drawing. The log-rank test was applied to compare overall survival difference between different groups. The ‘survival’ package was used for univariate and multivariate Cox regression analysis. Correlation heatmap were drawn using R packages ‘corrplot’. Kaplan-Meier estimates were used for survival analysis, with a two-sided log-rank test. The Student’s t-test was employed to compare two groups and ANOVA analysis was performed to compare multiple groups. *P* < 0.05 was considered as a statistical difference.

## Results

### Construction of natural killer cell-related gene signature

To characterize the Natural killer cell-related gene expression in gliomas, we examined the RNA-seq data of glioma patients from CGGA and TCGA datasets. We found 134 Natural Killer cell-related genes from immport. Subsequently, 397 genes belong to 18 GO pathway (GOBP_NATURAL_KILLER_CELL_ACTIVATION, GOBP_NATURAL_KILLER_CELL_ACTIVATION_INVOLVED_IN_IMMUNE_RESPONSE, GOBP_NATURAL_KILLER_CELL_CHEMOTAXIS, et al.) related to natural killer cell was collected from MSigDB. After removing the replication genes, 244 genes were left. Details on the Natural Killer Cell-Related genes are presented in Supplement Table 1 and Table 2. By combining the rank of *p* values of the univariate Cox regression analysis and Kaplan-Meier analysis, three genes (FDR < 0.05) were retained. A gene-based prognostic model was then established to evaluate the risk of each patient as described in the methods. Consequently, a three-gene signature was generated, and signature risk score was calculated as: risk score = (ULBP1*0.048) + (CD70*0.016) + (BID*-0.002). To validate this gene set, we also calculated patients’ risk scores of the CGGA cohort with the same regression Coeffs.

### Overall survival status of glioma patient based on gene signature risk score in CGGA and TCGA datasets

**Fig. 1:**
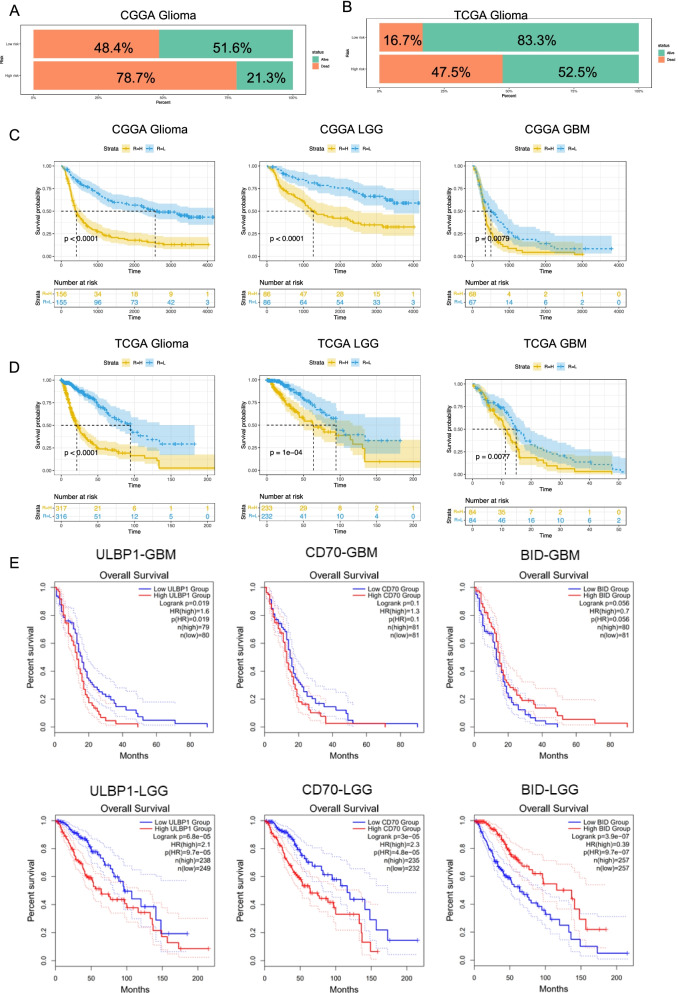
Prediction of outcome of the gene signature for patients based on the risk score. **A, B** Alive patients percentage was higher in low risk group than in high risk group in CGGA dataset and TCGA dataset. **C, D** Patients in high risk group had a significantly shorter OS than in low riskgroup in both of the CGGA databaset and the TCGA dataset. **E** The association between OS and genes used to generate risk score were demonstrated individually

Patients with different kinds of glioma were divided into two groups based on their median risk scores. Their percentage of alive patients was 51.6% in the low-risk group versus 21.3% in the high-risk group in the CGGA dataset (Fig. 1A). Similarly, alive patients were 83.3% in low-risk group versus 52.5% in high-risk group in the TCGA dataset (Fig. 1B). The Kaplan–Meier curve for the CGGA dataset showed that the high-risk patients had significantly shorter OS than low-risk patients in the glioma (Fig. 1C), WHO low-grade glioma, and GBM (Fig. 1C). The consistency of results were validated for the TCGA (Fig. 1D). The association between OS and genes used to generate risk score were demonstrated individually (Fig. 1E).

### Nature-killer cell gene signature is associated with clinicopathological features

**Fig. 2:**
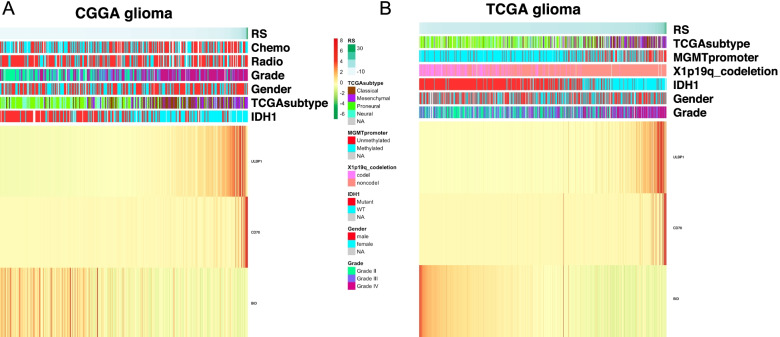
Nature-killer cell gene signature is associated with clinicopathological features in CGGA and TCGA datasets. **A** Heatmap of the correlation between risk score (RS) and clinicopathologic features in CGGA dataset. **B** Heatmap of the correlation between risk score (RS) and clinicopathologic features in TGGA dataset

**Fig. 3:**
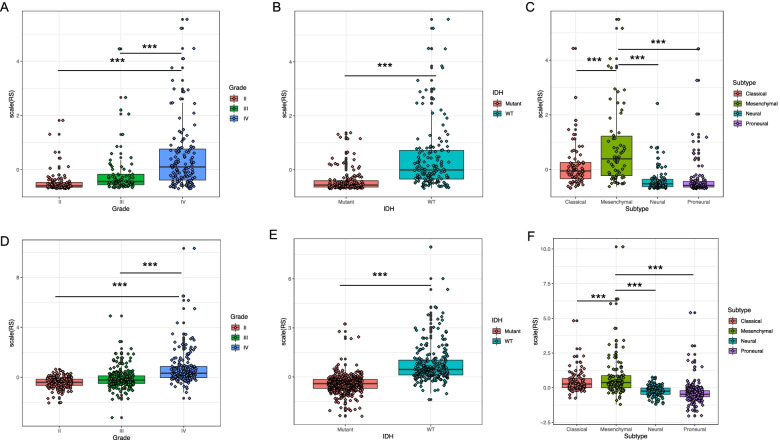
Nature-killer cell gene expression is correlated with clinicopathological features of gliomas. **A** The relationship of RNA expression of Natural-killer cell gene with glioma WHO. **B** IDH wild type have a higher expression level of NK related gene than IDH mutant type. **C**The relationship of RNA expression of Natural-killer cell gene with glioma subtypes. **D-F** Consistent results were also validated in the TCGA datasets

The heatmap showed that Nature-killer cell gene expression was correlated with WHO grade, IDH1 mutation, MGMT promoter methylation, 1p19q co-deletion, tumor subtype in glioma patients in CGGA (Fig. 2A) and TCGA datasets (Fig. 2B). Those data were also confirmed by Cox regression analysis (Table 1 and Table 2). We used the non-parametric Spearman correlation test to calculated the correlation between RS and clinical pathologic characteristics, and demonstrated that RS was significantly related with patients’ clinical characteristics, such as WHO grade, tumor subtypes, and IDH1 mutations (*p* < 0.001) in both two databases (Table 3 and Table 4). After mining the CGGA dataset, the RNA expression of Natural-killer cell gene was higher in high grade glioma than WHO grade II patients (Fig. 3A), higher in the IDH wild type than IDH mutant glioma (Fig. 3B). It was the highest expressed in the Mesenchymal group when compared with Classical, Neural and Proneural glioma types (Fig. 3C). These consistent results were also validated in the TCGA datasets (Fig. 3D-F). Moreover, univariate Cox regression and multivariate Cox regression of the signature of the natural killer-related genes were performed in the CGGA dataset (*p* < 0.001, univariate Cox regression; *p* < 0.05, multivariate Cox regression, Table 1). The independence of the clinical prognostic significance of the signature in glioma. The risk score showed significance in both univariate and multivariate Cox regression. Similar results were also validated in the TCGA dataset (Table 2). The patients with a high-risk score had a markedly higher mortality rate than those with a low-risk score in these two datasets. Meanwhile, with an increase in glioma grade, the risk score increased.

**Table 1:** Univariate and multivariate Cox regression analysis of clinical pathologic features for OS in CGGA

	CGGA cohort					
	Univariate analysis			Multivariate analysis		
Characteristics	*P*-value	HR	95% CI	*P-*value	HR	95% CI
Age	< 0.001	1.033	1.02–1.047	0.938	1.001	0.985–1.017
Gender	0.621	0.931	0.7–1.237	0.897	0.979	0.707–1.356
Grade	< 0.001	2.017	1.736–2.344	< 0.001	1.455	1.199–1.767
Subtype	< 0.001	1.631	1.428–1.862	0.007	1.281	1.069–1.535
IDH1	< 0.001	0.367	0.27–0.501	0.118	0.721	0.479–1.086
Radio	< 0.001	0.505	0.368–0.694	< 0.001	0.49	0.345–0.696
Chemo	0.001	1.658	1.219–2.255	0.098	1.343	0.947–1.905
Risk score	< 0.001	17.093	6.993–41.783	0.048	4.1	1.009–16.652

**Table 2:** Univariate and multivariate Cox regression analysis of clinical pathologic features for OS in TCGA

	TCGA cohort					
	Univariate analysis			Multivariate analysis		
Characteristics	*P-*value	HR	95% CI	*P-*value	HR	95% CI
Age	< 0.001	1.073	1.061–1.084	< 0.001	1.066	1.049–1.084
Gender	0.462	0.9	0.681–1.191	0.68	1.08	0.749–1.559
Grade	< 0.001	3.093	2.637–3.628	0.028	1.33	1.032–1.714
Subtype	< 0.001	2.004	1.765–2.275	0.11	1.194	0.961–1.484
IDH	< 0.001	8.561	6.228–11.768	0.17	1.616	0.814–3.21
MGMT promoter	< 0.001	0.228	0.139–0.376	0.026	0.508	0.28–0.921
1p/19q	< 0.001	2.964	2.17–4.047	0.5	1.165	0.748–1.816
Risk score	< 0.001	1.126	1.099–1.154	0.007	1.066	1.018–1.117

**Table 3:** The correlation between clinical pathologic features and RS in CGGA

Characteristics	Correlation coefficient	*P-*value
Gender	0.115	0.068
Grade	0.582	< 0.001
Subtype	0.583	< 0.001
IDH1	−0.609	< 0.001
Radio	−0.054	0.393
Chemo	0.195	0.002

**Table 4:** The correlation between clinical pathologic features and RS in in TCGA

Characteristics	Correlation coefficient	*P-*value
Gender	− 0.42	0.358
Grade	0.497	< 0.001
Subtype	0.606	< 0.001
IDH	−0.687	< 0.001
MGMT promoter	−0.434	< 0.001
1p/19q	−0.561	< 0.001

### GO and KEGG pathway analyses (gene functional characteristics related to risk scores)

**Fig. 4:**
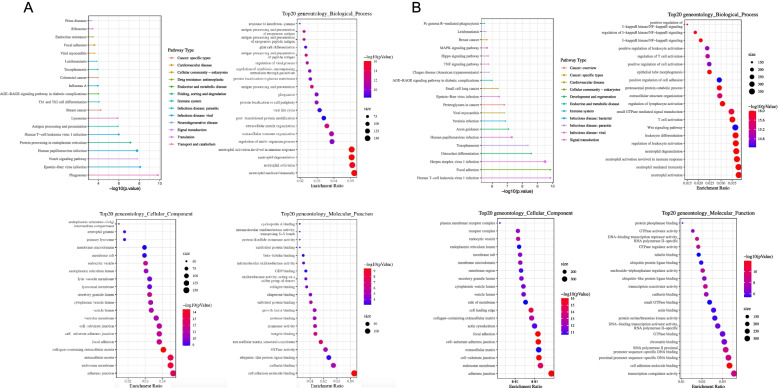
Gene functional characteristics related to risk scores. GO and KEGG analysis of differential genes between low- and high risk cases in two cohorts (**A** and **B**)

To investigate the function of NK cell-related genes in GBM cells, we analyzed different functional enrichment between low and high-risk cases. TOP 20 pathway type, biological process, cellular component, and molecular function were demonstrated by GO/KEGG enrichment respectively (Fig. 4A and B). The GO enrichment analysis showed the most enrichment pathway was phagosome; the most activated biological processes were neutrophil activation involved in immune response, neutrophil degranulation, neutrophil activation, neutrophil-mediated immunity; the cellular components were largely enriched in extracellular matrix, endosome membrane, and adherens junction; the most enriched molecular function was cell adhesion molecule binding (Fig. 4A). Similarly, the KEGG enrichment analysis confirmed that focal adhesion and Human T-cell leukemia virus 1 infection were the major activated pathways which were also enriched by GO analysis. The major biological process in KEGG were neutrophil-mediated immunity and neutrophil activation; the most enriched cellular component and molecular function were adherens junction, cell adhesion molecule binding and transcription coregulator activity, all of which was similar to the GO analysis (Fig. 4B). Taken together, these results indicated that the difference between low and high-risk score of NK cell-related gene signature were lines in immune-related adhesion, neurophil activation and T cell leukemia virus 1 infection.

### Correlation analysis between risk score (RS) and immune checkpoints/NK marker genes

**Fig. 5:**
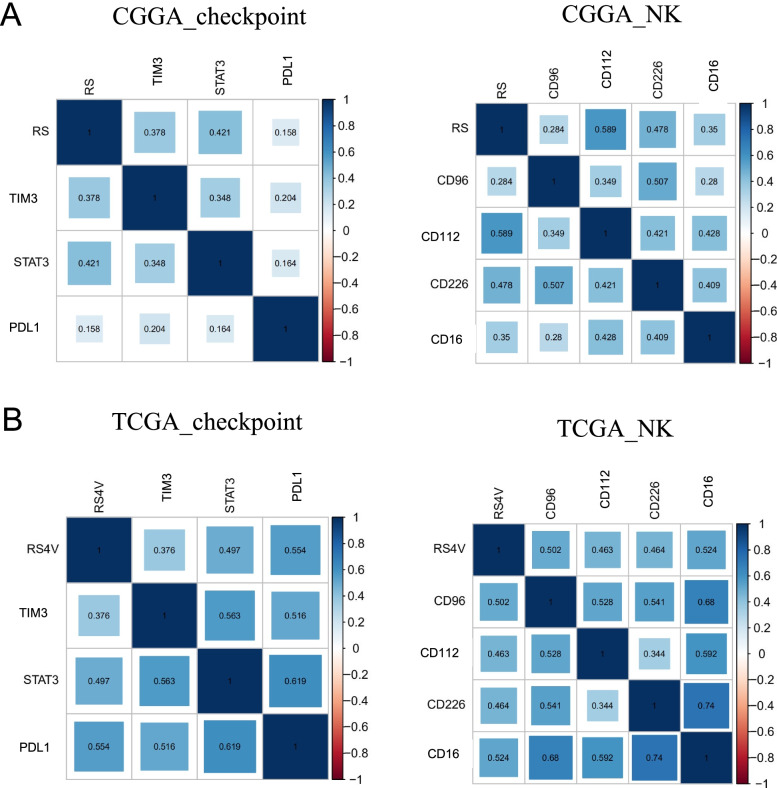
Comparison of difference in immune status between high-risk and low-risk groups. Corrlation heatmap showed correlation analysis between risk score (RS) and immune checkpoints/NK marker genes in CGGA **(A)** and TCGA **(B)** dataset

To understand the correlation between risk score (RS) and immune checkpoints/NK marker genes, we analyzed the data by correlation heatmap of co-expressed genes and found that gene signature risk score was related with PDL1, TIM3 and STAT3 in CGGA and TCGA datasets (Fig. 5A). As with the NK markers evaluated with the risk score, we demonstrated that risk score was correlated with CD16, CD226, CD96 and CD112. In summary, the Nature-killer cell gene signature expression is closed related to immune-related pathways and cancer immunotherapy process (Fig. 5B).

### Prediction of patient outcome based on the RS and immune checkpoint gene expression

**Fig. 6:**
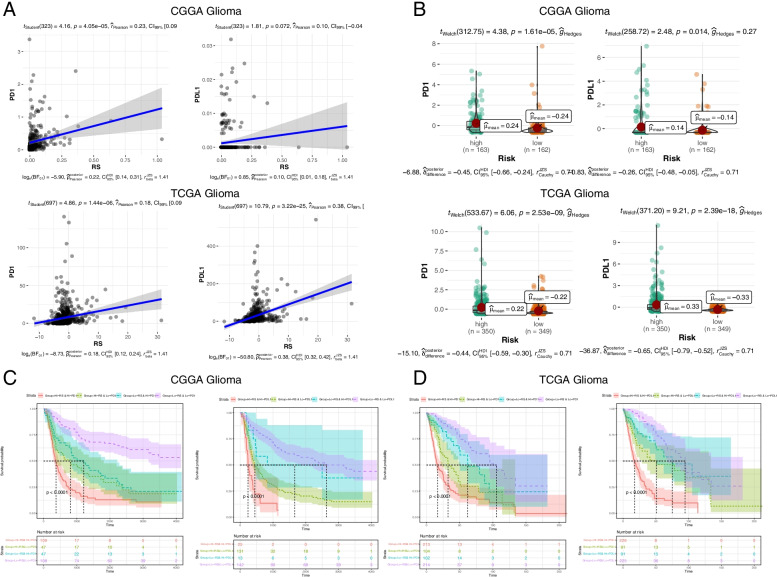
Prediction of patient outcome based on the RS and immune checkpoint gene expression. **A** Correlation coefficient of the RS and PD1/PDL1 gene expression. **B** The expression distribution of PD1/PDL1 in the high- and low-risk groups. **C** Overall survival curves of glioma patient stratified by the RS and immune checkpoint gene expression

To deeply figure out the status between the risk score, checkpoint gene expression and patient survival. We initially made a pearson analysis between PD1, PDL1 and risk score. As showed in Fig. 6A, the correlation coefficient of pearson analysis of PD1 and RS was 0.23 and 0.18 in CGGA and TCGA datasets respectively, the correlation coefficient of PDL1 and RS was 0.10 and 0.38 in CGGA and TCGA. These data indicated that RS has a tight relation with PD1/PDL1 expression (Fig. 6A). Then, we stratified the RS to low and high group and found that both PD1 and PDL1 were higher expressed in high-risk score group than the low-score group by mining both CGGA and TCGGA datasets (Fig. 6B). Lastly, we sorted out the data to four groups to better understand the RS, PD1/PDL1 and patient prognosis, Kaplan–Meier survival curves of OS among four patient groups showed that low-RS-low PD1 group as well as low-RS-low PDL1 had a better overall survival than other three groups (Fig. 6C). This data was verified by TCGA dataset (Fig. 6D). In summary, NK cell-related gene signature combined with PD1/PDL1 can be applied to predict patient’s prognosis, and low-RS-low-PD1/PDL1 patients showed better survival outcomes.

## Discussion

In this work, we investigated the association between NK cell-related gene signature and glioma. Firstly, we developed an NK cell-related signature and confirmed that it was closely associated with the overall survival of patients in the CGGA and TCGA datasets.

After screening, three genes are involved in NK cell biology and function. UL16 binding proteins (ULBPs) are natural killer group 2D (NKG2D) ligands which could hinder the activation of NK cells. Increased serum ULBP1 predicted reduced overall survival of hepatocellular carcinoma patients [30]. ULBP1 also interacted with NKG2D to improve survival of gastric cancer patients by induction of adaptive immunity [31]. ULBP2 functioned as a strong prognostic marker in malignant melanoma, p53-mediated increasing of cellular miR-34 levels to control ULBP2 expression. Those data indicated that tumor suppressors are also indirectly connected with ULBPs [32]. Elevated expression of ULBP3 was identified in a large amount of tumor cell lines and tumor tissues, it regulated the activity of NK cells against tumors and could be a prominent target for immunotherapy [33]. Taken together, ULBPs played a vital role in tumor biological function and patient overall survival.

CD70 belongs to the tumor necrosis factor (TNF) ligand family, and its only receptor CD27 is expressed on T cells and NK cells. Chronic costimulation of CD27-CD70 interactions can led to lethal T cell immunodeficiency [34]. In contrast to normal tissue, CD70 is expressed in brain tumor cells, especially gliomas and meningiomas [35]. CD70 might affect tumor progression directly, or indirectly by influencing the immune response. Wischhusen et al. identified that CD70-mediated apoptosis of immune effector cells may act as a novel immune escape pathway of malignant gliomas [36].

BH3-interacting domain death agonist (Bid), a pro-apoptotic member of the Bcl-2 protein family, encodes a death agonist and regulate apoptosis. There are both pro- and anti-apoptotic proteins in Bcl-2 family and these proteins can bind to each other to form a complex network of homo- and hetero-dimers. All the memebers belong to anti-apoptopic Bcl-2 group could be able to act as oncogenes. Such as overexpression of Bcl-2 caused by chromosomal translocation lead to an increased incidence of follicular lymphoma. In turn, pro-apoptotic Bcl-2 members are tend to inhibit tumors’ occurrence. For example, mutation of Bax increases tumourigenicity of several cancers [37, 38]. BID is unique in the Bcl-2 family since it links the extrinsic and intrinsic apoptotic pathway [39]. After the death receptors is activated, BID is cleaved by caspase-8 into an N-terminal p13 and a C-terminal truncated BID (tBID) which could transfers from cytoplasm to mitochondria and induces the release of cytochrome C, resulting in apoptosis [40]. Bid was also found to play crucial roles in inflammation and innate immunity by interplaying with nucleotide-binding and oligomerization (NOD) 1, NOD2, and the IκB kinase (IKK) complex [41].

Subsequently, we found that this signature could distinguish the clinical and molecular features of gliomas, including WHO grade, TCGA subtype, IDH mutational status, 1p19q co-deletion and MGMT promoter methylation. Based on the differentially expressed genes of the risk score, GO enrichment results indicated that the major difference between high -risk and low-risk score focused on virus infection, immune system, neuropil mediated immunity, cell adhesion. KEGG enrichment results also confirmed that the above immune- anti-apoptotic related functional and signaling pathways. These results suggested that low-risk score NK cells related signature activated immune system through immune cells infiltration and cell adhesion.

Immunotherapy has shown encouraging benefits for many cancer types. In the current study, we found RS was tightly related to NK CD markers, CD96 and TIGIT together with the co-stimulatory receptor CD226 form a pathway which could enhance the immune response [42]. In addition, Sun H et al. [43] found that human intratumoral CD96+ NK cells are functionally exhausted and patients with higher intratumoral CD96 expression exhibit poorer clinical outcomes. CD 16, also known as FcγRIII, is a differentiation molecule found on the surface of natural killer cells, which antibodies, such as cetuximab could mediate apoptosis by CD16 receptors after the recognition [44]. NK cells are large granular lymphocytes of the innate immune system which can directly lyse infected or tumor cells [45, 46]. Increasing evidence demonstrated that NK cells played a vital role in killing GBM by different approaches like KIR, CD16, IFN-γ, TNF-α, NIKG2D, TGF-β, CAR-NK and NK-exosomes [17, 46]. Clearly, NK cell-based immunotherapy is more and more attractive for GBM treatment [2]. Our study showed RS was interconnected with CD16, CD226, CD96 and CD112, which could activate NK cells to kill glioma cells to achieve prolonged survival.

Immune checkpoints were the most promising treatment targets against cancer. Thus, we investigated the correlation between RS with PDL1 and TIM3, which showed low RS linked high PDL1, high RS linked with low TIM3. Laterly, we combined checkpoints and RS to predict the overall survival of glioma patients, as expected, low-RS-low-PD1/PDL1 group gained the most prolonged OS. Therefore, high RS is recognized as an unfavorable feature of glioma. PD-1, an extensively studied immune checkpoint receptor, is expressed on immune cells to limit harmful immune responses and prevent overactive immune-driven pathology [47]. But this immune regulation mechanism could led to prolonged chronic disease courses such as chronic viral infections and cancer since dysregulated inhibitory receptor expression on immune cells prevents the elimination of tumors and viruses. Furthermore, immunotherapy that block the inhibitory receptors PD-1 and CTLA-4 has been successful in the tratment of several cancers [48].

In summary, our research provided important prognostic resources based on NK cell-related gene signature in glioma. It will contribute to the exploration of NK cell research to promote novel immunotherapy against glioma.

## Supplementary Information

**Additional file 1 **: **Supplement Table 1**. NK Cell related Genes from IMMPORT.

**Additional file 2 **: **Supplement Table 2**. NK Cell Related Molecular Signatures in Database.

## Data Availability

All the data websites were confirmed to be available online. The RNA sequencing data and corresponding clinical information were downloaded from the TCGA (https://portal.gdc.cancer.gov/) and CGGA (http://www.cgga.org.cn). The immune-related gene list was observed from the IMMPORT website (https://www.immport.org/). Futher inquiries for the datasets generated and analyzed during the current study are available from the corresponding author on reasonable request.
